# Viscous Fingering in Multiport Hele Shaw Cell for Controlled Shaping of Fluids

**DOI:** 10.1038/s41598-017-16830-3

**Published:** 2017-11-30

**Authors:** Tanveer ul Islam, Prasanna S. Gandhi

**Affiliations:** Suman Mashruwala Advanced Microengineering Laboratory, Department of Mechanical Engineering, Indian Institute of Technology Bombay, 400076 Powai, Mumbai India

## Abstract

The pursuit of mimicking complex multiscale systems has been a tireless effort with many successes but a daunting task ahead. A new perspective to engineer complex cross-linked meshes and branched/tree-like structures at different scales is presented here. Control over Saffman-Taylor instability which otherwise randomly rearranges viscous fluid in a ‘lifted Hele-Shaw cell’ is proposed for the same. The proposed control employs multiple-ports or source-holes in this cell, to spontaneously shape a stretched fluid film into a network of well defined webs/meshes and ordered multiscale tree-like patterns. Use of multiple ports enables exercising strong control to fabricate such structures, in a robust and repeated fashion, which otherwise are completely non-characteristic to viscous fingering process. The proposed technique is capable of fabricating spontaneously families of wide variety of structures over micro and very large scale in a period of few seconds. Thus the proposed method forms a solid foundation to new pathways for engineering multiscale structures for several scientific applications including efficient gas exchange, heat transport, tissue engineering, organ-on-chip, and so on. Proposal of multi-port Hele-Shaw cell also opens new avenues for investigation of complex multiple finger interactions resulting in interesting fluid patterns.

## Introduction

Plentiful existence of branched tree-like patterns in nature is an outcome of natural iterative process, over the millennia, towards the perfection of such designs. These branched structures therefore have been of interest to be used for various applications. Some examples include, microfluidic mixers and networks^1,2^, capillary pump^3^, synthetic leaf^4^, non-transparent solar electrodes^5^, and most importantly, heat exchangers^6,7^, mass transporters^8,9^, and vascular systems^2,10–12^. Tree-like structure with integrated source and sink segments have been demonstrated to be mechanically strong and at the same time energy efficient in flow and mass transport than any other design configuration^13,14^. Vascular systems also exhibit tree-like patterns at higher scales with extremely branched constructs that transform them into a web of interconnected micro capillaries^15,16^. These micro-capillaries are less in the form of tree-like and more as complex web of interconnected networks. *In vitro* models of such micro networks are of tremendous help to the field of artificial organ growth, response of tissues to various in-development treatment drug, curing diseases, cell sorting and many other tissue engineering applications^17^.

Among many techniques of fabricating such complex capillary-networks^18^, well-defined engineered micro arrays/channel-networks^19,20^ have shown momentous advancement in bio-applications^21–23^. Fabrication techniques presented in the literature are limited either in scalability^23^ or in time efficiency or controllability^24^. We propose a non-conventional, spontaneous, time-efficient, lithography-less method of shaping fluid into well-defined meshes and tree-like structures with source and sink segments by exercising control over Saffman-Taylor instability in a lifted Hele-Shaw cell. In Hele-Shaw cell^25^ a thin viscous fluid film, sandwitched between two parallel plates, when displaced by a less viscous fluid (normally air) develops Saffman-Taylor fingering instability^26^. In a “lifted Hele-Shaw cell” a variant of the same interface instability is induced by lifting apart the cell plates, sucking-in low viscous air as long air-columns/fingers from its peripheries^27^. Lifting of cell plates, done either angularly^28^ or parallely^29,30^, leaves a random and normally short-lived network of high-viscous fluid branches on both the cell plates. The pattern layout could substantially be altered by micro-patterning anisotropies on the cell slides^27,31–33^. We recently proposed use of yield-stress fluids in a lifted Hele-Shaw cell to retain the structures permanently^34^ and also the use of pits as anisotropies to control the Saffman-Taylor instability for shaping/fabricating fractal-like structures^31^.

In this paper, we first characterize the effect of a single hole (termed as source-hole) in lifted Hele-Shaw cell. We further propose to use multiple such sources-holes/ports as controlling units, for the first time to the best of our knowledge, to shape fluid in the desired patterns mentioned above. Our previous work^31^ demonstrated controlled shaping of fluid into branched patterns using control over fluid-air interface offered by blind pits that cross the interface. Additional control was exercised by manipulating depth of these pits. In contrast, in this paper, we use fundamentally different scientific principle of using multiple source-holes on the cell plate (forming what we term as multiport Hele-Shaw cell) inside stretched fluid interface. Thus the proposed control in this case is exercised by relative placement of these source-holes as against the limited control offered by depth of pits only at boundary in the previous case. Controlled branched patterns obtained in this paper, hence show distinct difference in terms of connected branch ends as against free branch ends in our previous work^31^. For example, we demonstrate in this paper shaping of fluid into ordered mesh patterns at two different scales and a replica of leaf venation. Thus, the proposed philosophy of using source-holes establishes solid foundation for shaping fluid into families of desired patterns at multiple scales. The shaping/fabrication process, irrespective of the structure scale, completes in a period of few seconds and the structures thus obtained are solidified followed by casting them into hollow channel-networks using an elastomer or a hydrogel as per the application requirement.

## Results

Shaping of fluid into desired structures involve exercising control over highly noise sensitive viscous fingering process in a Hele-Shaw cell. Section-1.1 introduces fundamental concepts behind the proposed shaping of fluid by controlling viscous fingering and identifies process parameters. Section-1.2 discusses development of recipe for the desired shaping by using the results of characterization of process parameters. Structure fabrication/shaping, based on the control recipe thus evolved, is demonstrated in Section 1.3. Discussion in Section-2 summarizes the work, followed by methods in Section-3 explaining materials and experimental procedures used.

### Proposed control using source-holes

**Figure 1:**
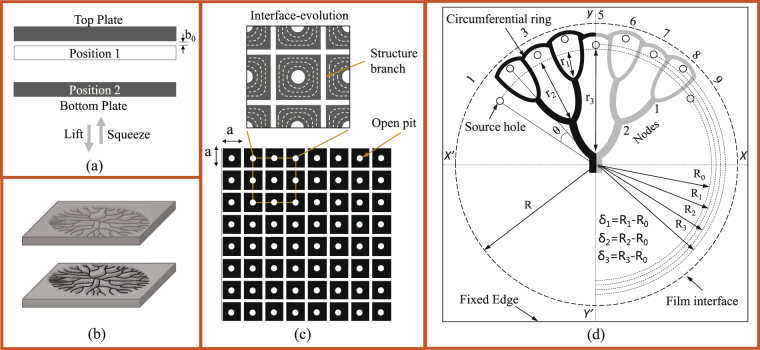
Schematic representation of (**a**) Lifted Hele-Shaw cell with parallel separation wherein the bottom plate squeezes the fluid against a top fixed plate to a final thickness of *b*_0_ and radius *R* (position 1). Separation of plates is carried out while maintaining parallelity between the two cell plates (position 2). (**b**) Isometric representation of Hele-Shaw cell and the type of branched pattern that evolves, in the absence of controlling source-hole, on both cell plates as mirror images of each other. (**c**) Layout of ports/source-holes on one of the cell plate for fabricating a regular square arrayed structure with each cell size *a* × *a*. Enlarged section shows evolution of the interface (uniform interaction among the fingers) in lifting process giving rise to ordered structure branches. (**d**) Layout of source-holes on a cell plate and the type of controlled Cayley tree structure that would evolve from such a layout. Source-holes are placed at a radius of ${R}_{0},{R}_{1},\mathrm{...}{R}_{n}$ from the center wherein ${\delta }_{n}={R}_{n}-{R}_{0}$ with all the source-holes equally apart from each other by an angle *θ*. The inner most source-holes are at a radius of *R*_0_ and the radius to which the fluid film interface is stretched is *R*. Non-uniformity (waveness) in circumferential ring is an exaggeration, of actually near-circular ring formed, so as to represent the source-hole positions at different ${R}_{n}^{^{\prime} }s$. In experiments, magnitude of *δ*_*n*_ nedeed to shape the fluid into a tree-like pattern is very small (~*μm*).

Random pattern evolution in a lifted Hele-Shaw cell (Fig. 1(a)) is a two stage process-initiation (onset of Saffman-Taylor instability) and progression (shielding mechanism) of air finger. Initiation is the destabilization of stretched fluid film interface into a random waveform, when a low viscous air tends to rushes-in as soon as lifting commences. The length and width to which each wavelet/finger would grow, compared to others, depends on the relative inception (in terms of time and position) of each wavelet. This relative growth due to finger-finger interaction, known as shielding mechanism^35^, is what patterns the fluid into a highly disordered/random structure, graphically shown in Fig. 1(b). During cell lifting process, due to shielding mechanism, the number of fingers that supersede others reduce exponentially^36^ whereas their size in length and width continues to increase^37^.

We propose shaping of fluid using multiple-ports or source-holes in lifted Hele Shaw cell (LHSC) now termed as multiport lifted Hele Shaw cell (MLHSC). Introduction of multiple-ports/source-holes in a cell plate allows the controlled initiation of air fingers into the stretched film at many desired points. Such initiation of fingers is explored to completely transform the process for shaping fluid into well-defined mesh patterns as against random branched patterns formed in lifted Hele-Shaw cell. For obtaining well-defined mesh patterns, for example, source-holes are placed at the centroid of each cell-unit or each square as schematically shown in Fig. 1(c). Fingers emerging out of such multiple-ports are made to interact uniformly as schematically shown in Fig. 1(c). Uniform interaction is assured if all the fingers initiate simultaneously. To achieve simultaneous initiation of fingers, ports are sealed until separation begins to avoid liquid rise in ports. Controlled initiation should be followed by no tip-splitting/destabilization of fingers so as to avoid uncontrolled extra-branch formation and to have a uniform interaction. Finger-splitting/destabilization for a fluid with surface tension *σ* and viscosity *τ*_0_ is a function of lifting velocity *V* and cell parameters *b*_*o*_ and *R*. In what follow in Section 1.2, a modified non-dimensional number involving these parameters is proposed to carry out the characterization of finger splitting event. Although the pattern evolution process is explained here for uniformly spaced port arrays, it can be seen easily that the concepts can be extended to predict pattern evolution with non-uniform arrays of ports. Note here that because of multiple ports pressure inside film gets redistributed as against parabolic increase, from periphery towards cell center, in a cell without ports.

As another example to demonstrated effectiveness of proposed control method, we consider shaping of fluid into tree-like patterns. For controlled shaping of fluid into tree-like pattern source-holes are arranged along the circumferential region, just inside the stretched film interface as shown in Fig. 1(d), as an example. Fingers, as long air-columns, emerging out of these source-holes are made to undergo controlled non-uniform interaction by having source-holes placed at different distances (*R*_*n*_) from the film center. Non-uniform interaction implies the unequal growth of fingers for shaping the fluid into an ordered tree-like pattern. Controlled non-uniform interaction via initiation-manipulation is again possible only for fingers which propagate while maintaining their stability i.e., which do not undergo tip-splitting mechanism^27^. To ensure this the lifting velocity, is always maintained below a threshold above which random tip-splitting events are observed.

### Recipe for shaping fluid

**Figure 2:**
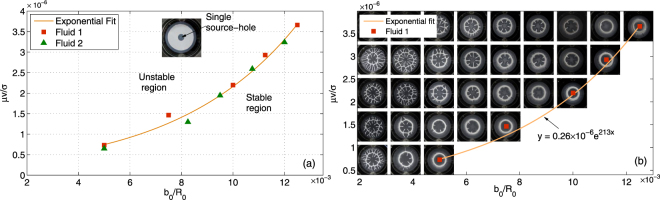
Source-hole characterization. (**a**) Stabilization of finger emerging from a single central source-holes is shown here for increasing *V* and *b*_0_. Data is plotted between two non-dimensional quantities *μ* *V/σ* and *b*_0_/*R*_0_ and experiments were performed for two fluids with large difference in their rheological parameter (see Supplementary Fig. 1(a)). Radius *R* = 20 *mm* is kept constant for all cases. Inset image shows an intermediate lifting stage where a circular finger (air) penetrating from a single central source-hole displaces the fluid. The image also shows retreating of fluid from the outer interface. (**b**) Pictorial representation of how the finger destabilization process evolves before it reaches a stable point for different *V* and *b*_0_ values. The pattern left on the cell plates, after the lifting process is complete, shows number of finger formations decrease as *b*_0_ increases (left to right) until no finger destabilization happens thus shaping the fluid into a smooth circular ring (last image of all rows). Lifting velocity *V* was varied from 2.5 *μm/sec* to 12.5 *μm/sec* (step increase of 2.5 *μm/sec*) and minimum *b*_0_ value was taken to be equal to ${50}^{\pm 2}\mu m$ (step increase of 25 *μm*). All images are from the experiments carried out for fluid-1 and the images for fluid-2 are shown in Supplementary Fig. 2.

Towards development of recipe for shaping fluid into desired arrayed structures, parameters influencing the process are identified and characterized first. As mentioned in Section 1.1 fabrication involves uniform interaction of fingers with no destabilization, the characterization of parametric dependencies of the finger destabilization process is thus presented in Fig. 2(a,b). Evolution of a single finger, emerging from a source-hole present at the center of a plate, with variation in parameters is shown in the figure. We plot the non-dimensional number *μV*/*σ* against cell parameters *b*_0_/*R* and a curve is fitted to separate region with unstable evolution from that with stable. Validation of fitted curve is confirmed by studying the destabilization for two fluids (Fluid 1 and Fluid 2) with a contrasting difference in *σ* and *τ*_0_. The fluids used are shear-thinning fluids which follow Herschel-Bulkley model ($\tau ={\tau }_{0}+k{\gamma }^{n}$) as shown in Supplementary Fig. 1(a). Although viscosity of the fluids vary with shear rate, it is equitable to consider the value of *μ* at *γ* ≤ 1 for calculating capillary number *μV*/*σ* as the velocity of stable fluid interface at the highest (*V* = 12.5 *μm*/*s*) is very very low (<1 *mm*/s) as shown in Supplementary Fig. 1(b). For a given *V* (say, bottom row in Fig. 2(b) where *V* = 2.5 *μm*/*s*) the number of branches formed (tip-splitting events) is less frequent and decreases as *b*_0_ increases. For evolution without destabilization the fluid gets collected into a circular ring as can be seen in the last image of each row in Fig. 2(b). At low lifting velocity (*V*) it is found that both the outer and inner interface stabilizes at lesser *b*_0_. All the arrayed patterns could be fabricated with interface-stability dependable parameters laying on or to the right of curve in Fig. 2(a,b)-stable region.

Having mentioned characterization results and fundamental concepts in proposed control, the following simple recipe is proposed for fabrication of arrayed structures:

1. Given arrayed pattern, determine the centroids of each unit cell as locations for source-hole anisotropies.
2. For multiport Hele Shaw cell with anisotropies determined in step 1 use parameters in stable region of Fig. 2(a,b) to obtain the patterns.

**Figure 3:**
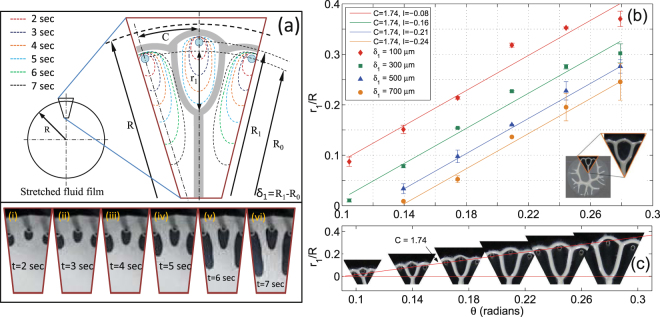
Single finger-finger interaction. (**a**) Schematic diagram showing initiation and progression of two outer and one central finger from source-holes placed at a raduis of *R*_0_ and *R*_1_ respectively, where *R*_0_ < *R*_1_. Images i–vi are frames taken from a video-record of actual experiment showing the controlled non-uniform interaction. Corresponding to six stages (image i–vi) of the process, schematic diagram also represents six interface evolution stages. At time *t* = 4 sec all the fingers have grown to almost equal lengths (orange line) however outer fingers are ahead of central one as they had initiated from source-holes closer to the cell center (*R*_0_). At time *t* > 4 outer fingers grow faster in size as they over-taken the central finger, getting more area to spread out, eventually shielding the center finger to a limited size. Fluid left between the fingers is shaped into long branches with a merging point (node) at the tip of central finger. Fluid, which had initially spread beyond the source-holes recollects itself into a circumferential branch. (**b**) The figure shows that with increase in *θ*, *r*_1_ increases linearly with constant slope of 1.74 for all $\delta ^{\prime} s$. Also as delta increases *r*_1_ decreases for the same *θ*. (**c**) Pictorial representation, from actual structures fabricated, of variation in *r*_1_/*R* against *θ*. Shapes formed here are form a Hele-Shaw cell with *b*_0_ = 50 *μm*, *R*_0_ = 15 *mm*, *θ* = 10° and *δ*_1_ = 100 *μm*.

For shaping of fluid into desired tree-like pattern, as shown schematically in Fig. 1(d), interaction of a single finger with two adjacent/outer-fingers is first characterized. Simulation results presented in^35^ demonstrated shielding of a finger by using numerically advanced initial positions to fingers adjacent to it. Further widening of these side fingers in a continuously decreasing area of spread, while progressing towards the center, completely stops/shields the central fingers to a certain length. Inspired by this and considering simultaneous finger initiation (as mentioned earlier) from all ports in our case, we propose relative placement of ports to affect shielding process. Specifically, we place ports for initiation of two outer fingers at a smaller radius *R*_0_ relative to radius *R*_1_ where port for initiation of central finger is placed (${R}_{1}-{R}_{0}={\delta }_{1},{\delta }_{1}\mathrm{ > 0}$, see Fig. 3(a)). Variation of center finger length *r*_1_ (see Fig. 3(a)) against angular distance (*θ*) between two adjacent source-holes for different $\delta ^{\prime} s$ is shown in Fig. 3(b,c). In agreement with the simulation results^35^ the normalized finger length *r*_1_/*R* follow a linear relation against *θ* given by *r*_1_/*R* = *Cθ* + *I*, where *C* is slope and *I* the intercept. Graph in Fig. 3(b) show that with increase in *δ*_1_, the value of *C* remains constant (=1.74) where as *I* decreases linearly. With *r*_1_/*R* showing a reliance on both *θ* and *δ*_1_ (represented by changing *I*), its variation against *δ*_1_ is separately plotted in Supplementary Fig. 3. Note here that any finger length *r*_*n*_ is measured as a distance traveled by that finger beyond the circumferential-line passing through source-holes placed at *R*_0_ as shown in Figs 1(d) and 3(a).

The characterization of single *δ*_1_ can further be used to conceive recipe for several desired tree-like patterns. As an example we consider a Cayley-tree type structure^38^ with co-ordination number 3 and upto three generations as shown in Fig. 1(d) as a desired structure. Here the source-holes 2,4,6 and 8 are placed at maximum radius *R*_3_ so that fingers initiate from them travel smallest length *r*_1_. Source-hole 3 and 7, which give rise to 2nd generation fingers, travel a distance of *r*_2_ while shielding all 1st generation fingers are thus placed at *R*_2_ such that *R*_2_ < *R*_3_. Finger from source-hole 5 terminates at *r*_3_ before shielding 1st and 2nd generation fingers along its way thus setting a position for source-hole 5 at *R*_1_ such that *R*_1_ < *R*_2_. Finally finger 1 and 9 have to be ahead of all other fingers so that they can supersede all other fingers before shielding finger 5 at *r*_3_ and are therefore places at a minimum distance of *R*_0_. The recipe can be generalized as: In order to obtain an *nth* order Cayley tree with coordination number 3, source-holes generating fingers ${r}_{1},{r}_{2},{r}_{3}\mathrm{...}{r}_{n}$ should be placed in such a way so that ${R}_{0} < {R}_{1} < {R}_{2}\mathrm{... < }{R}_{n}$ where ${R}_{n}-{R}_{0}={\delta }_{n}$. Similar recipes can be developed for other tree-like patterns (using same principles).

### Shaping of fluid into desired patterns

#### Well defined meshes

**Figure 4:**
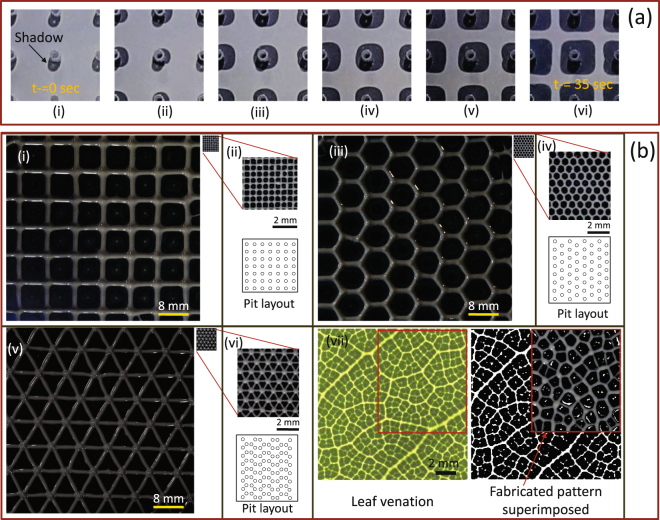
Mesh patterns. (**a**) Evolution of fingers initiating at almost the same time and uniform interaction of central finger equally with fingers from all four sides as the time progresses (image i–vi) is shown here. Fingers initiate with a circular shape (image ii & iii) and gradually transform into rectangular shapes (image iv–vi) as the interaction effect comes into play. Shadow of source-holes created due to light source incorrectly displays a deformed view of expanding finges in image ii & iii. Parameters set were *b*_0_ = 50 *μm*, *a* = 5 *mm*, and *V* = 4 *μm/s*. (**b**) Triangular, square, and hexagonal structures mimicking complex vascular systems are fabricated at two different scales. Image i, iii, and v show the geometrical patterns fabricated at a much higher scale wherein each branch is about 8 mm in length. These macro pattern were fabricated with *b*_0_ = 125^±10^ *μm*. Micro-meshes with the same layout as macro structures are also shown in these figures to demonstrate the fabrication at two different scales. Image ii, iv, and vi show magnified micro-patterns with each branch length less then 1 mm and *b*_0_ = 50^±5^ *μm*. Images in vii show a leaf venationn^39^ and its grayscale-form to highlight the venations pattern as a set of polygons. Enclosed part is fabricated to mimic the venation pattern in its exact form by having source-hole layout determined by locating the centroid of each polygon.

Having developed recipes for arrayed mesh and tree like patterns with corresponding characterization of parameters, we next demonstrate experimentally the effectiveness of the proposed fabrication at micro and higher scales. Although we demonstrated fabrication of structures with overall feature dimensions of 8 *mm* and 900 *μm* and Cayley tree structures (with co-ordination number 3) upto three generations, the control strategy can be extended to fabricate even higher generations and over much larger areas. Multiport Hele-Shaw cell is used to fabricate mesh’s with different linkage configurations viz. square, triangular and hexagonal. Micro structures with branch dimensions in micro domain and on other hand same structures with much bigger branch dimensions were considered for fabrication. To fabricate a structure, say square mesh shown schematically in Fig. 1(c), all source-holes need to be placed at the center of each structure unit (say, square) so that fluid is equally displaced by a penetrating finger shaping the fluid into a square mesh. Finger from a single port interacting uniformly with other surrounding fingers thus shaping the fluid into a square layout is shown in Fig. 4(a). To fabricate a mesh with some other branch configuration (say a triangular or hexagonal mesh), source-holes are to be placed at the center of each geometric unit. To demonstrate the potential of fabricating at micro and higher scales Fig. 4(b) shows structures on a single scale (i, iii and v) and on a magnified view (ii, iv and vi). Branch length and height in case of micro structures (ii, iv and vi) are 900 and 35–40 *μm* where as in macro structures (i, iii and v) is 8 and 0.5 *mm* respectively. The proposed method can not only fabricate arrayed symmetric structures (Fig. 4(b)) but also mimick relatively random patterns for example leaf venation (Fig. 4(b)(vii)). Source-holes were placed at the centroid of each polygon formed by the venation pattern. Fabricated and actual pattern is superimposed to match the two venation patterns.

#### Tree-like pattern

Recipe proposed in Section 1.2 is now used to fabricate and further characterize tree-like structures with all end branches connected by a circumferential ring thus forming an integrated source and sink system. Any of the three source-holes (say source-hole 1, 2 and 3 in Fig. 1(d)) could be considered for fabrication of first generation structures. Normalized finger distance *r*_1_/*R* of only first generation structure fabricated, as explained in Fig. 3(b,c), follows a linear relation with *θ*. Slope *C* = 4 from simulation^35^ differ hugely form the experimental value of *C* = 1.74 whereas intercept *I* = 0 is very close to the value of $I=-0.08\simeq 0$ form experiments when *δ*_1_ = 100 *μm*. This huge variation in *C* is attributed to the assumptions made in the simulations about the non-existence of *σ* and weak finger-finger interaction set-in by providing extra amplitude to two end fingers in the form of numerical noise. A strong finger-finger interaction is set in all the experiments by choosing a high value of *δ*_1_.

**Figure 5:**
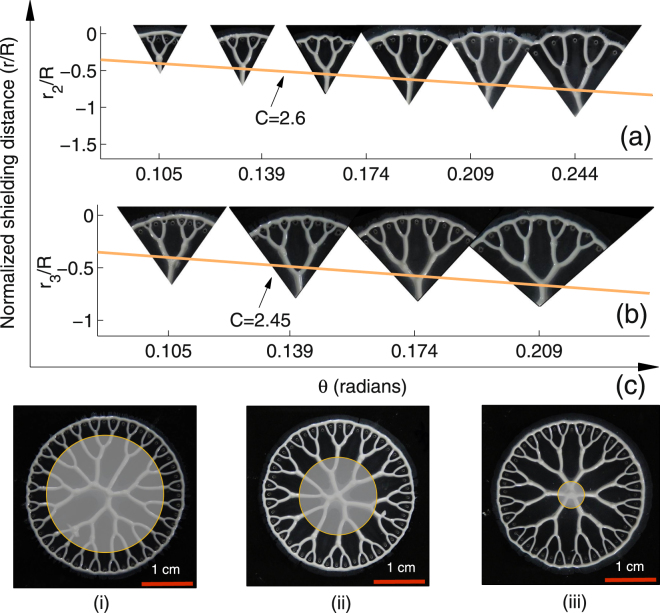
Tree-like patterns. Pictorial representation of increase in node distance (*r*_*n*_) with increasing *θ* for (**a**) second generation structure where ${\delta }_{1}=200\,\mu m,$ and *δ*_2_ = 400 *μm* (**b**) third generation structure with ${\delta }_{1}\mathrm{=200}\,\mu m$, ${\delta }_{2}=300\,\mu m,$ and ${\delta }_{3}=400\,\mu m$. (**c**) Images show a controlled non-uniform interaction of fingers initiating over the entire interface. Image-i show only the controlled first generation structures i.e, branches emerging out of the shaded circular portion (reference circle) divide only once before they merge with the circumferential ring. Image-ii shows second and image-iii shows third generation structures over 360° interface. All the fingers in image-iii are made to under go controlled interaction for their entire lengths thus producing a cayley-tree structure in its actual/ideal form. Parameters set for shaping these structures are, *b*_0_ = 50 *μm*, *R* = 15 *mm θ* = 7.5° and *V* = 4 *μm*/*s*.

Similar to the first generation *r*_1_/*R* plots, normalized curves *r*_2_/*R* and *r*_3_/*R* for second and third generation structures were obtained from the experiments and plotted over the actual structures shown in Fig. 5(a) and (b) respectively. Structures shown here are obtained by controlled shaping of a section of the stretched fluid film with increasing *θ*. Any of the five source-holes (1–5 or 5–9) and all the 9 source-holes form Fig. 1(d) could be considered for fabrication of second and third generation structures respectively. Controlled shaping of first, second, and third generation structures over the entire interface of 360° shown in Fig. 5(c) demonstrates a completely controlled non-uniform interaction using sealed source-holes. Multiscale nature of controlled structures fabrication is demonstrated in Supplementary Fig. 4. Placement of source-holes to shape the fluid into mesh and tree-like structures discussed separately so far could be combined together to fabricate an integrated structure as demonstrated in Supplementary Fig. 5. Video record of experiments of controlled non-uniform interaction for fabrication of third generation structure over the entire 360° interface and uniform interaction of fingers to shape the fluid into a mesh structure is shown in Supplementary Video 1.

## Discussion

This paper presented simple yet elegant control over Saffman-Taylor instability leading towards development of time and cost effective recipes that shape viscous fluids into patterns widely observed in the nature at multiple scales. Specifically two distinct classes of patterns were demonstrated: a mesh patterns with desired layouts and an ordered branched pattern with smoothly varying branch thicknesses (Supplementary Fig. 4). Use of multiport Hele-Shaw cell was an epiphany to shape fluids into layouts which are radically unlike those in conventional Hele-Shaw cell. Fabrication of highly ordered mesh structures (square, triangular and hexagonal layouts) demonstrates a very high degree of control over the Saffman-Taylor instability otherwise known to produce uncontrolled branched patterns. Fabrication of layouts precisely mimicking, with very ease and robustness, the flow transporting structures in plant leaves and other such systems redefines this process as exemplary method to bio-mimic structures. Apart from controlling robustly the finger-finger interactions, shaping of fluid into a Cayley-tree type structures with an important feature of connected end branches was demonstrated. Cayley-tree type structures characterized upto order three, have branch ratios (${r}_{n}/{r}_{n+1}$) in ranges that bear likeness to various structures exiting in nature. With appropriate fluid selection the minimum branch thickness (first generation branches) in these controlled structures can go down to few microns. Homogeneity of fluid used, source-hole and cell plate roughness, precision in plate separation are however the factors affecting the controlled fabrication at exceedingly smaller film thicknesses. At higher film thicknesses the process is relatively less affected by these factors and thus controlled fabrication can be carried out at larger scale. Fabricated structures when cast into channels networks find many application such as: in tissue growth, as efficient heat exchangers, mass transportation, capillary pumps, cell sorting and migration, artificial vasculature ans so on.

The proposed control, leading to robust repeatable fabrication would help establish otherwise difficult to obtain, correlation between simulated and experimental patterns in this domain. Proposal of multi-port Hele-Shaw cell also opens new avenues for investigation of complex multiple finger interactions resulting in interesting fluid patterns. Although we considered only one type of fluid (ceramic suspension with small yield stress) in the present paper, investigation using proposed ideas with different fluids (for example viscoelastic) can further open up interesting fluid dynamics and applications.

## Methods

### Experimental setups

A setups to work as parallely lifted Hele-Shaw cell, shown in Supplementry Fig. 6, was designed to operate at high precision. As the fingering process is highly sensitive to the existence of minuscule gradient in plate parallelity, a self-paralleling mechanism was used to restrict the gradient within few microns. To provide the squeeze and lifting force, motorized translation stage (MTS-90115–1–01, from Holmarc) operated via micro positioning controller (HO-MPC-3L, from Holmarc) was used. Position of lower cell plate, while squeeze and lifting process, was monitored using a linear position encoder (RGH25F, from Renishaw). A dSPACE’data acquisition card’ (ds1104) was used to interface linear position encoder with operating PC.

### Fluid preparation

A fluid with a small yield stress property is prepared from a monomer HDDA (1, 6 Hexanediol diacrylate, by Sigma Aldrich) added with alumina (Almatis, CT3000, Mean particle size 0.5 microns, refractive index 1.7), 4wt.% Benzoin ethyl ether (BEE, as photo-initiator) and 2.5 wt.% Phosphate ester (PE, as surfactant). Alumina powder is incrementally added while the ceria stabilized zirconia balls were used to homogenize the fluid on a pot mill. The prepared fluid is a photo-resist ceramic suspension with viscosity, surface tension, and yield stress values dependent on the volume fraction of alumina added. Modular compact rheometer (Anton paar Physica MCR301) and Wilhelmy plate method was used to measure the fluid viscosity-profile and surface tension, respectively.

### Controlling source-holes

Using a CNC-micro drilling machine source-holes were drilled on one of the cell plate for fabricating the structures. Diameter of source-holes in the present study is kept equal to 0.5 *mm* for all the experiments.

### Design of experiment

A measured quantity of the fluid at the center cell plate is first compressed to required thickness (*b*_0_) and radius (*R*). Source-holes, machined using the CNC machine, are sealed in advance form one side to prevent the penetration of fluid into them while squeezing the fluid. Before the separation process begins the sealing is removed to let the source-holes act as sources of low-viscous fluid. For tree-like pattern fabrication, compressed fluid film is allowed to spread radially beyond the source-holes by a maximum distance of around 1.5 mm. A delay of 30 seconds is introduced to stabilize the fluid film, followed by plate separation at constant velocity of 4 *μm/s*. Constant separation velocities in mesh structure fabrication case varies from 2.5 to 12.5 *μm/s* for different experiments. Complete experimental process is recorded by a camera (18 MP NIKON S9400) at 30 fps. Structure features like the finger lengths ${r}_{1},{r}_{2},\mathrm{...}{r}_{n}$ are measured using Image-J software and 3D profile of individual branches is mapped by white light interferometry (WLI) technique (MSA 500, Polytech, Germany).

## Electronic supplementary material

Supplementary Movie

Supplementary File
