# Supplementary material for: Viscous Fingering in Multiport Hele Shaw Cell for Controlled Shaping of Fluids
Source: Sci Rep. 2017 Nov 30;7:16602. doi: 10.1038/s41598-017-16830-3 (PMC5709420; doi:10.1038/s41598-017-16830-3)
Supplement: Supplementary file 2 — Supplementary File [file 41598_2017_16830_MOESM2_ESM.pdf]

Viscous Fingering in Multiport Hele Shaw Cell for Controlled Shaping of Fluids

Tanveer ul Islam[1] **and Prasanna S. Gandhi**[1,*]

[1]Suman Mashruwala Advanced Microengineering Laboratory, Department of Mechanical Engineering, Indian Institute of Technology Bombay, 400076, India
[*]gandhi.iitb@gmail.com

Supplementary Movie. 1. The supplementary movie shows controlled shaping of fluid into a Cayley-tree structure and a triangular micro-mesh pattern.

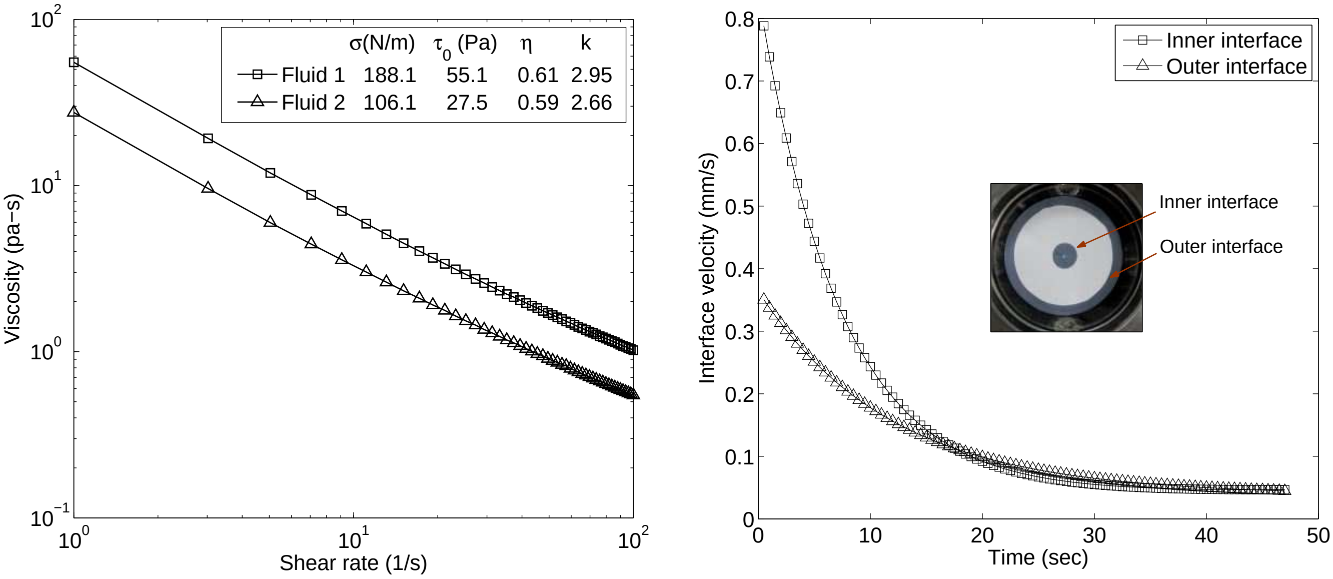

(a) (b)

Supplementary Fig. 1. (a) Graph shows shear-thinning nature of the two fluids which follow a Herschel–Bulkley model where $\tau = \tau_0 + k\gamma^n$. The model parameters, for the two fluids, are listed in legend table. (b) Graph shows velocity variation of inner and outer interface for a single source-hole during lifting phase. Data is plotted for highest $V(12.5\mu m/s)$ where as b_0 is chosen such that the two interfaces are stable. Although viscosity of the fluids vary with shear rate, however it is equitable to consider the value of μ at $\gamma \leq 1$ for calculating the non-dimensional number $\mu V/\sigma$ as the velocity of stable fluid interfaces at highest V $(12.5\mu m/s)$ is very very low $(< 1mm/s)$ as shown here.

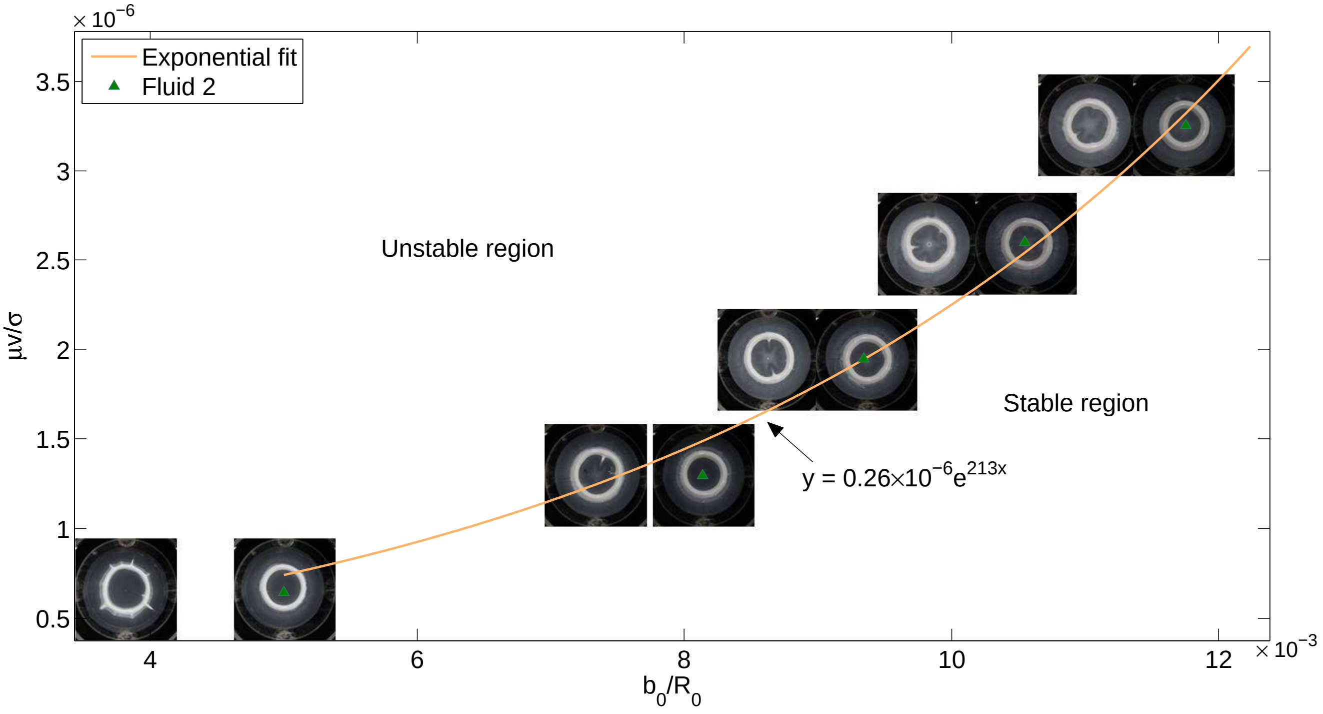

Supplementary Fig. 2. Figure shows pictorially the final pattern left, in case of fluid-2, on the plates with single source-hole. The fitted curve, as plotted in Fig.2(a), passes through the points where both the interface had propagated stably and formed a smooth ring. All the structures lying in the stable region will form a smooth ring as no finger-splitting/ destabilization occurs. The figure also shows one image in each case which falls in the unstable region and thus have some extra branches formed due to destabilization.

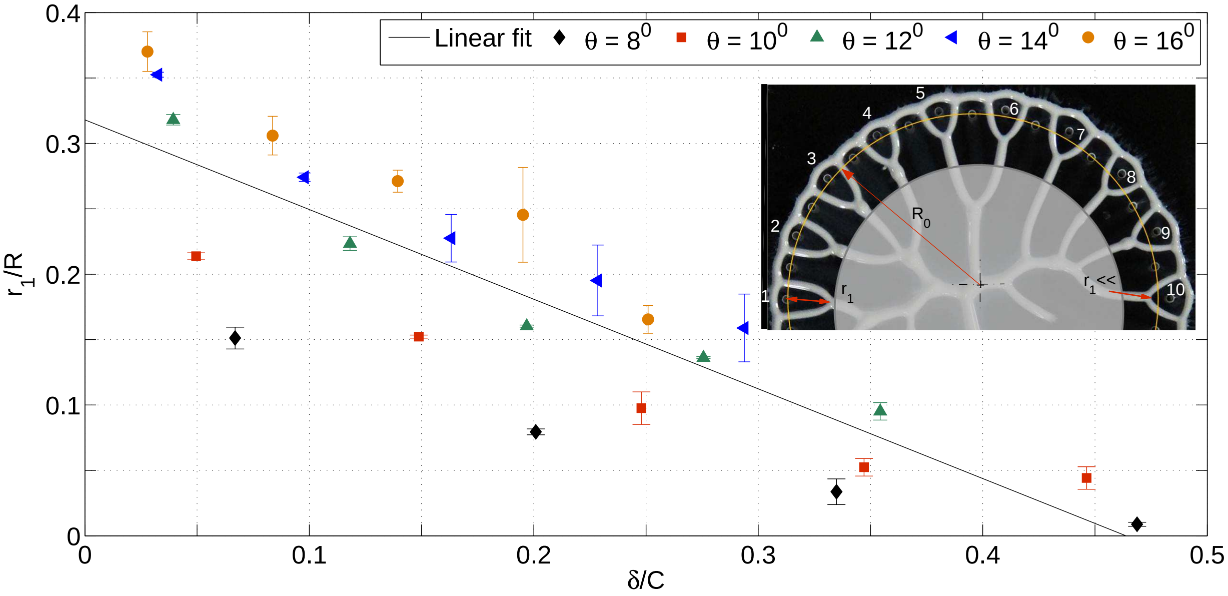

Supplementary Fig. 3. (Limit on δ) Graph shows that decrease in r_1/R reaches a vanishing point when the ratio δ_1/C is within the range 0.4-0.6. Linear fit represents the convergence of r_1/R towards a δ_1/C value of $0.5^{\pm 0.1}$ for fingers emerging from source-holes at various $\theta' s$. Inset image shows decrease in r_1 as δ_1 increases form $100\mu m$ (source-hole no. 1) to $1000\mu m$ (source-hole no. 10), with a step increase of $100\mu m$. Each source-hole numbered from 1-10 is surrounded by two source-holes placed at a constant radial distance of $R_0 = 15mm$ (reference circle marked) and at equal angular separations. Fingers emerging from numbered source-holes (1-10) are shielded by fingers from source-holes placed at a radial distance of $R_0 = 15mm$.

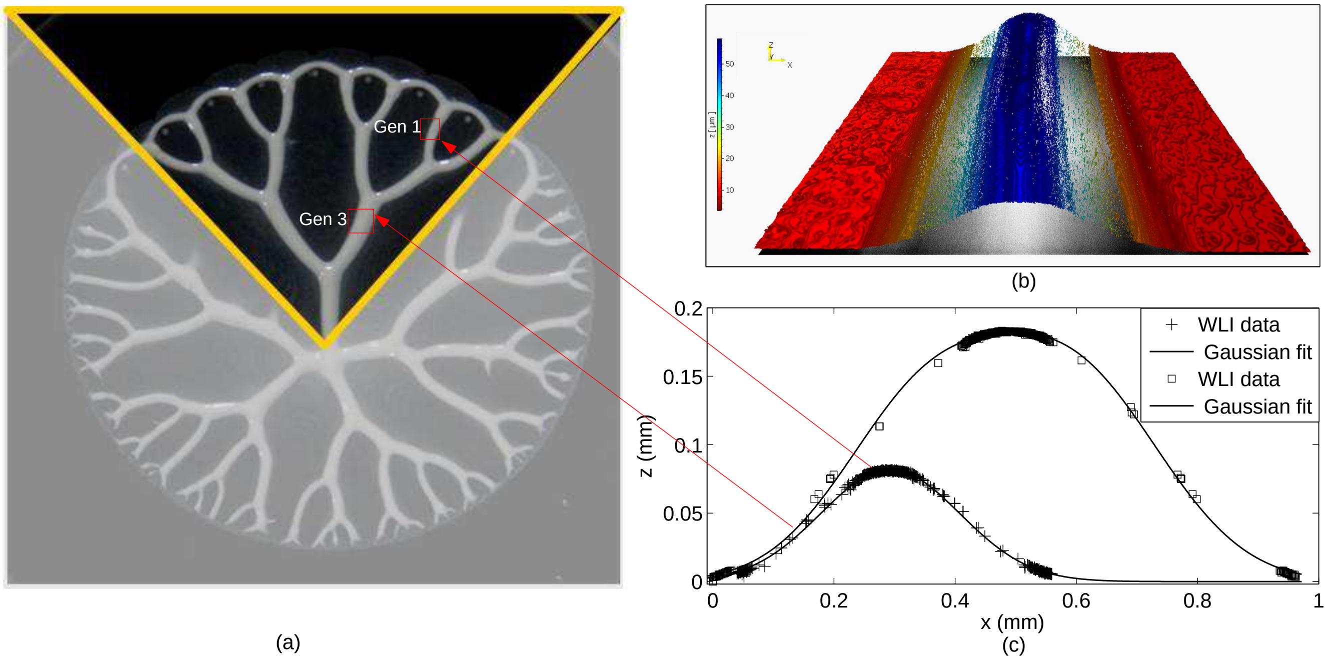

Supplementary Fig. 4. (a) Third-generation structure with 1st and 3rd generation branch indicated. Her $b_0 = 40\ \mu m$ and $R = 30\ mm$. (b) Three dimensional profile of a branch obtained through White Light Interferometry (WLI). (c) Graph shows the cross-sectional profile of 1st and 3rd generation branch as indicated by the arrows. The branch size increases smoothly from first to higher generations.

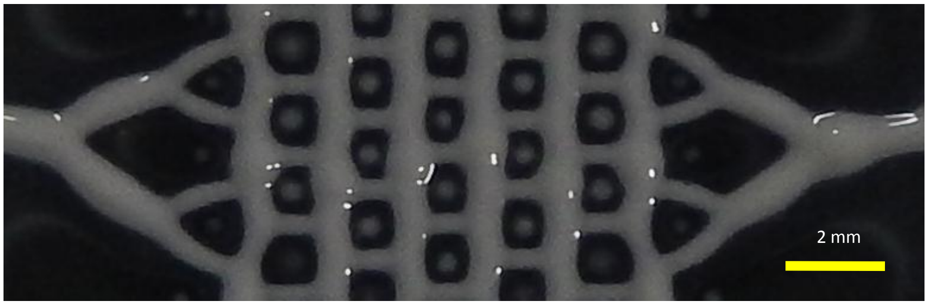

Supplementary Fig. 5. Demonstration of applying both the fabrication techniques to fabricate a combined tree-like and mesh structure.

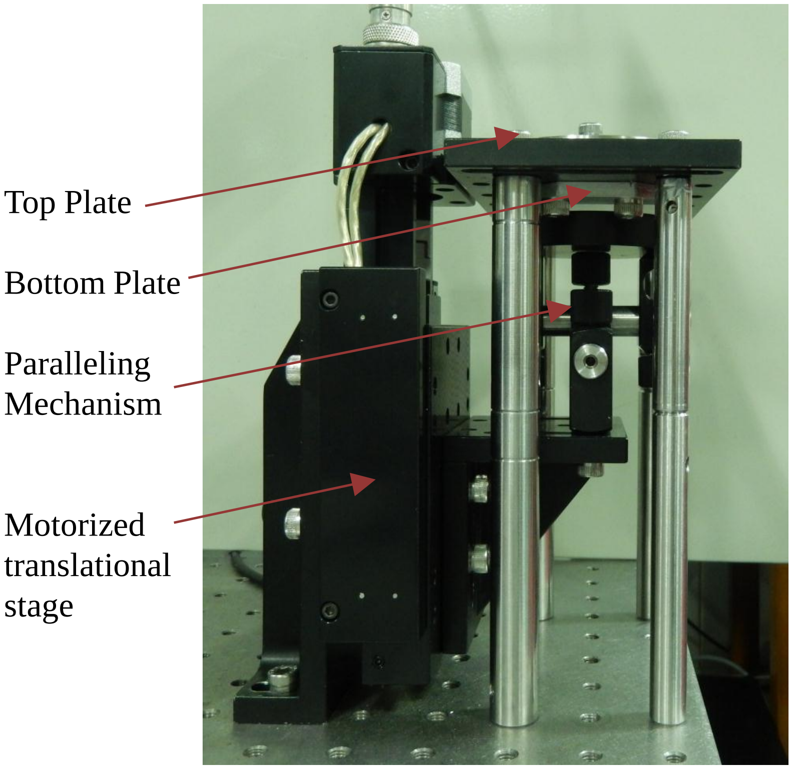

Supplementary Fig. 6. Experimental setups is designed to operate as parallel Hele-Shaw cell where a motorized translational stage is used for fluid squeeze and plate lifting at constant velocities. An angle-bracket holds the paralleling mechsnim aligning the bottom plate held highly paralell to the top plate. The top plate is held fixed between two metal rings as the sequeeze and lifting is achieved by actuation only the bottom plate. A camera is mounted at the top to get the visual information during the cell operation. An encoder (not visible in image) is attached to moniter the bottom plate position.
